# Use of Preventive Care Services and Hospitalization Among Medicare Beneficiaries in Accountable Care Organizations That Exited the Shared Savings Program

**DOI:** 10.1001/jamahealthforum.2021.4452

**Published:** 2022-01-07

**Authors:** Yajuan Si, Nicholas Moloci, Sitara Murali, Sarah Krein, Andy Ryan, John M. Hollingsworth

**Affiliations:** 1 Survey Research Center, Institute for Social Research, University of Michigan, Ann Arbor; 2 University of North Carolina at Chapel Hill; 3 Dow Division of Health Services Research, Department of Urology, University of Michigan, Ann Arbor; 4 Department of Internal Medicine, University of Michigan, Ann Arbor; 5 Department of Health Management and Policy, University of Michigan, Ann Arbor

## Abstract

**Importance:**

Thirty percent of Medicare accountable care organizations (ACOs) in the Shared Savings Program (SSP) have exited within 5 years of joining. Absent the potential for shared savings, exiting ACOs may choose to divest from costly resources needed to support population health, worsening clinical quality for beneficiaries aligned to these organizations.

**Objective:**

To examine the associations of SSP exit with clinical quality delivered to Medicare beneficiaries.

**Design, Setting, and Participants:**

This retrospective cohort study was conducted between 2019 and 2020 using national Medicare claims data from a 20% random sample of beneficiaries. A total of 1 713 237 beneficiaries were aligned with an SSP ACO at some point between 2012 and 2016. Distinction was made between those for whom the ACO to which they were aligned exited the SSP and those whose ACO stayed in the program. By comparing exiting ACOs with those that stayed in the SSP, changes in the quality of care that a beneficiary received before and after the aligned ACO exited the SSP were evaluated. Whether findings associated with exit varied with respect to the number of years after exit was also examined.

**Exposures:**

Exiting the SSP and the number of years after exit.

**Main Outcomes and Measures:**

Receipt of annual preventive care services and hospital utilization.

**Results:**

Among the cohort of 1 713 237 beneficiaries (mean [SD] age at enrollment, 75.20 [7.96] years), 998 511 (58.3%) were female, 126 123 (7.4%) were Black, and 1 482 823 (86.6%) were White. Exiting the SSP was associated with statistically significantly lower rates of annual glycated hemoglobin A_1c_ testing (odds ratio [OR], 0.74; 95% CI, 0.68-0.81), low-density lipoprotein cholesterol testing (OR, 0.86; 95% CI, 0.76-0.97), and all diabetes complication screening (OR, 0.90; 95% CI, 0.81-0.97) for beneficiaries with diabetes. The exit was not associated with rates of hospital utilization in terms of emergency department visits and 30-day readmission after SSP exit. The associations with exit depended on the length of time since contracts ended. For example, the baseline rate of annual glycated hemoglobin A_1c_ testing was 89.8% (95% CI, 89.5%-90.1%) but fell to 86.9% (95% CI, 85.9%-88.0%) and 86.8% (95% CI, 85.0%-88.5%) in years 1 and 2 after exit, respectively, but then rose to 91.9% (95% CI, 85.3%-98.5%) in year 3.

**Conclusions and Relevance:**

In this cohort study of Medicare beneficiaries, SSP exit was associated with modest declines in clinical quality. These findings are timely given recent SSP changes that could accelerate program exit.

## Key Points

**Question:**

How is the exit of an accountable care organization (ACO) from the Medicare Shared Savings Program (SSP) associated with clinical quality delivered to beneficiaries, and does the association change over time after exit?

**Findings:**

In this cohort study of more than 1.7 million Medicare beneficiaries, SSP exit was associated with considerably lower rates of preventive service use, though not associated with rates of hospital utilization. These associations differed depending on how far removed an ACO was from SSP participation, where the reductions in clinical quality were most prominent in the first 2 years after exit.

**Meaning:**

Observations of declines in clinical quality after ACO exit from the SSP are important given recent changes to the SSP that could accelerate program exit.

## Teaser

This cohort study examines the associations between exit of accountable care organizations from the Medicare Shared Savings Program and clinical quality delivered to beneficiaries.

## Introduction

The Centers for Medicare & Medicaid Services (CMS) launched the Medicare Shared Savings Program (SSP) in 2012. Under this alternative payment model, groups of physicians, hospitals, and other health care organizations contract with CMS as accountable care organizations (ACOs), taking responsibility for the quality and total cost of care for a defined population. If participating ACOs are able to reduce their annual spending below a benchmark, they stand to share a percentage of the savings with CMS.^1^ Importantly, the SSP is a strictly voluntary program, and participating ACOs can exit without penalty. Recent empirical work shows that 30% have exited within the first 5 years of joining the program.^2^

The associations of program exit with care quality received by beneficiaries, who had been ACO aligned, are not clear. On the one hand, if an organization is an ACO “in name only,” then its exit likely has minimal influence. On the other hand, emerging data suggest that a substantial number of SSP ACOs have engaged in meaningful care redesign,^3^ but this engagement is costly. In fact, SSP ACOs spend an estimated $1.5 million annually on health information technology, data analytics, and care-coordination services to support population health.^4^ Absent the potential for shared savings, an exiting ACO may choose to divest from these resources. Consequently, the beneficiaries who were aligned with it could experience poorer clinical quality, especially under a hastened transition.

To determine the associations of SSP exit with clinical quality, we analyzed claims data from a 20% national sample of Medicare beneficiaries. First, we identified those who were aligned with an SSP ACO at some point between 2012 and 2016, distinguishing between beneficiaries for whom the ACO to which they were aligned exited the SSP and those whose ACO stayed in the program. We then assessed a variety of clinical quality measures among these beneficiaries throughout the study interval. Finally, we estimated changes in the clinical quality that a beneficiary received before and after the aligned ACO exited the SSP and time-varying associations regarding the number of years after exit.

## Methods

### Overview

After linking medical claims from Medicare beneficiaries with SSP ACOs, we considered 2 approaches with multiple periods to estimate changes in the quality of care delivered to ACO-aligned beneficiaries before and after the ACO to which they were aligned exited the SSP. The first approach used the indicators of SSP exit as the exposure variable to measure the overall exit association, and the second approach used indicators of the number of years since SSP exit as the exposure variable to measure the time-varying associations after the contract ends. Considering the possibility of cohort effects and violations of model assumptions, we then modified the sample inclusion and model specification and implemented a comprehensive set of sensitivity analyses to achieve a robust evaluation. The institutional review board at the University of Michigan determined that the study was exempt and participant consent was not needed owing to use of secondary data without identifiable information. This report has included the required items following the Strengthening the Reporting of Observational Studies in Epidemiology (STROBE) reporting guideline.

### Data Sources and Study Population

For this analysis (conducted between 2019 and 2020), we used claims data from a 20% national sample of Medicare beneficiaries after the launch of the SSP (2012-2016). Among those with continuous fee-for-service enrollment throughout a study year, we analyzed their paid claims appearing in the carrier, Medicare Provider Analysis and Review, and outpatient Research Identifiable Files (RIFs). We distinguished between beneficiaries aligned with ACOs exiting the SSP from those in organizations that stayed in the SSP with the SSP health care professional–level RIF. We excluded beneficiaries who did not receive any primary care services during a given year, as well as Medicare Advantage beneficiaries, because they were ineligible for ACO alignment according to the SSP final rule. We also excluded beneficiaries in ACOs that exited the SSP at some point during the study period but who were then aligned to another ACO that stayed in the program. The beneficiaries could switch ACOs in the SSP, but we kept the same assignment if the assigned ACOs ended the SSP contract. The primary analysis compared 2 groups of beneficiaries: (1) those aligned to an ACO that stayed in the SSP and (2) those aligned to an ACO that exited the SSP between 2013 and 2015.

We conducted a complete case analysis, assuming that any missing data were missing at random. Because different outcomes involved subsets of the sample, we fit separate models for them (eg, preventive care services only applied to beneficiaries 18-75 years of age with diabetes). Given the large sample, we do not expect substantial bias introduced by excluding cases with missing data.

### Exposure

We had 2 exposures of interest: (1) a time-varying binary indicator of SSP exit and (2) a count of the number of years after SSP exit. The binary indicator distinguished beneficiaries aligned to an ACO that exited the SSP between 2013 and 2015 and those in SSP ACOs. Because SSP ACOs could exit from their contract with CMS at any time, we distinguished exiting organizations by the time in years since their contract ended. For a given year, we could classify an ACO that was: (1) in the SSP, (2) 1 year out from its exit, (3) 2 years out from its exit, or (4) 3 years out from its exit. This differentiation allowed us to use the unbalanced data structure and evaluate whether the associations with exit were time varying.

### Primary Outcomes

At the beneficiary level, we evaluated several claims-based measures of clinical quality for the primary outcomes. These included receipts of preventive care services among beneficiaries 18 to 75 years of age with diabetes on an annual basis (glycated hemoglobin A_1c_ [HbA_1c_] and low-density lipoprotein [LDL] cholesterol testing, eye examinations, and all diabetes testing), as well as measures of hospital utilization among all beneficiaries (emergency department [ED] visits and all-cause 30-day readmissions among hospitalized beneficiaries).^5^ We selected these measures because they were well within the control of an organization. In addition, they were included in SSP contracts.^6^

### Covariates

From the Medicare Master Beneficiary Summary File, we assessed a variety of patient characteristics, including age, sex, race and ethnicity (Black, White, and other, including Asian, Hispanic, Native American, and other or unknown race) using the CMS Beneficiary Race Code, dual eligibility (for Medicare and Medicaid), and whether a beneficiary had end-stage kidney disease (ESKD). We evaluated a beneficiary’s level of comorbidity using the hierarchical condition category (HCC) methodology.^7^ With the SSP health care professional–level RIF and the Leavitt Partners ACO database,^8^ we also examined organizational differences between ACOs based on their number of primary care physicians, leadership structure (physician led, hospital led, or physician-hospital partnership), and size (the number of aligned beneficiaries and primary care and specialist physicians).

### Statistical Analysis

With the beneficiary-year serving as the unit of analysis, we fit a series of logistic regression models for each of the binary primary outcomes: *Υ_itk_*, where *Υ_itk_* equaled 1 if beneficiary *i* in ACO *k* received the care or the event occurred at year *t*; otherwise, *Υ_itk_* equaled 0. We considered a nonlinear trend with time to examine for differential changes in clinical quality before and after the ACO to which a beneficiary was aligned exited the SSP. The full model specification was as follows:



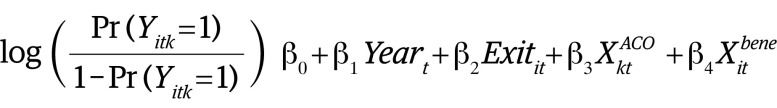



Here, Pr(*Υ_itk_*_ _=_ _1) is the probability of receiving the care or the event occurring. The covariate *Year_t_* was the calendar year indicator, and *Exit_it_* was the exposure variable. In the first set of models (M1), *Exit_it_* represented whether the ACO to which beneficiary *i* was aligned stayed in the SSP (*Exit_it_^M^*^1^ = 0) or exited the SSP (*Exit_it_^M^*^1^ = 1) at year *t*. In the second set of models (M2), *Exit_it_* represented whether the ACO to which beneficiary *i* was aligned stayed in the SSP (*Exit_it_^M^*^1^ = 0), exited the SSP for 1 year (*Exit_it_^M^*^2^ = 1), exited the SSP for 2 years (*Exit_it_^M^*^2^ = 2), or exited the SSP for 3 years (*Exit_it_^M^*^2^ = 3) at year *t*. The covariates *Χ_kt_^ACO^* represented the aligned ACO organizational characteristics previously described at year *t*. The adjusted covariates *Χ_ikt_^bene^* for beneficiary *i* included age, sex, race and ethnicity, dual eligibility, ESKD status, and the 79 HCC indicators at year *t*.

To account for potential correlations between beneficiaries cared for within the same ACOs, we calculated robust standard errors.^9^ The model with the unbalanced observational data had a few statistical, yet unverified, assumptions: (1) no cohort effects among ACOs joining the SSP in different years, (2) different groups of ACOs presented parallel trends, and (3) the performances were the same between pre-exit ACOs and ACOs staying in the SSP.

Finally, we performed a series of sensitivity analyses to test the robustness of the findings (eMethods in the Supplement). We performed all analyses using Stata, version 15.0 (StataCorp). We used Wald tests for odds ratio (OR) estimates in the logistic regression models, Pearson χ^2^ tests for testing different distributions of categorical covariates across groups, and Student *t* tests for continuous covariates in their distributional comparison between groups. All of the statistical tests were 2 tailed, with the probability of type 1 error set at *P* = .05.

## Results

The total cohort of 1 713 237 individuals (mean [SD] age at enrollment, 75.20 [7.96] years) included 998 511 (58.3%) female beneficiaries, 126 123 (7.4%) Black beneficiaries, 1 482 823 (86.6%) White beneficiaries, and 104 291 (6.1%) beneficiaries from other racial groups (Asian, Hispanic, Native American, and other or unknown race). In total, there were 3 695 038 ACO-aligned beneficiary-year observations from 2012 to 2016, 161 838 of which were observations made after an aligned beneficiary’s ACO exited the SSP. Table 1 summaries 1 594 009 beneficiaries aligned to ACOs that stayed in the SSP, with 119 228 aligned to exiting ACOs. Generally speaking, beneficiaries aligned to exiting ACOs vs those staying in SSP were more likely to be 70 years and older (81.6% vs 75.2%), Black (8.0% vs 7.3%), have ESKD (0.9% vs 0.7%), have dual eligibility status (12.2% vs 10.3%), and have a higher number of comorbid conditions (average HCC values: 1.85 vs 1.66) (*P* < .05). Table 1 also shows organizational differences between ACOs that stayed in the SSP (n = 312) and exited (4 ACOs exited in 2013, 28 exited in 2014, and 49 exited in 2015). The ACOs that exited SSP vs stayed in, on average, tended to have fewer aligned beneficiaries (7658 vs 12 928), as well as fewer specialist (94 vs 316) and primary care physicians (70 vs 170) (*P* < .001); they were also more likely to be physician-led organizations (65.7% vs 41.7%; *P* = .002).

**Table 1.** Beneficiary and Organizational Characteristics Among Accountable Care Organizations (ACOs) That Stayed in vs Exited the Medicare Shared Savings Program (SSP)

Characteristic	No. (%)		*P* value
	ACOs in the SSP	ACOs that exited the SSP^a^	
**Beneficiary level**			
Beneficiary-year observations	3 533 200	161 838	NA
Unique beneficiaries	1 594 009	119 228	NA
Sex			
Female	929 117 (58.3)	69 394 (58.2)	.57
Male	664 892 (41.7)	49 834 (41.8)	
Age, y			
66-69	857 939 (24.8)	29 813 (18.4)	<.001
70-74	876 920 (24.8)	43 175 (26.7)	
75-79	675 568 (19.1)	33 735 (20.8)	
≥80	1 104 773 (31.3)	55 115 (34.1)	
Race and ethnicity			
Black	116 601 (7.3)	9522 (8.0)	<.001
White	1 379 986 (86.6)	102 837 (86.3)	
Other^b^	97 422 (6.1)	6869 (5.8)	
No. of 79 HCC indicators, mean (SD)	1.66 (1.90)	1.85 (2.05)	<.001
ESKD	24 133 (0.7)	1413 (0.9)	<.001
Dual eligibility in Medicare and Medicaid	362 959 (10.3)	19 698 (12.2)	<.001
**ACO level**			
Unique ACOs	295	67	NA
Leadership structure^c^			
Hospital led	69 (23.4)	8 (11.9)	.002
Physician led	123 (41.7)	44 (65.7)	
Hospital-physician partnership	103 (34.9)	15 (22.4)	
No. of beneficiaries covered, mean (SD)	12 928 (16 523)	7658 (5935)	<.001
No. of PCPs, mean (SD)	170 (194)	70 (92)	<.001
No. of specialists, mean (SD)	316 (501)	94 (169)	<.001

Abbreviations: ESKD, end-stage kidney disease; HCC, hierarchical condition category; NA, not applicable; PCPs, primary care physicians.

^a^ Four ACOs exited in 2013, 28 exited in 2014, and 49 exited in 2015.

^b^ The Other category includes Asian, Hispanic, Native American, and other or unknown race, as defined by the Centers for Medicare & Medicaid Services’ Beneficiary Race Codes.

^c^ The leadership structure is missing for 17 ACOs in the SSP and 14 ACOs that exited.

Tables 2 and 3 summarize results from the 2 main multivariable models. The first model estimated the association between the measures of clinical quality and SSP exit, and the second model estimated the time-varying associations with SSP exit. After controlling for the patient- and organization-level differences described above, the first model showed that beneficiaries with diabetes who were aligned to an ACO that exited the SSP had lower odds of receiving annual HbA_1c_ testing (OR, 0.74; 95% CI, 0.68-0.81), LDL cholesterol testing (OR, 0.86; 95% CI, 0.76-0.97), or all diabetes complication screening (OR, 0.90; 95% CI, 0.81-0.97) following program exit than beneficiaries with diabetes aligned to an ACO that stayed in the program. Beneficiaries aligned to an exiting ACO following program exit tended to have higher rates of ED utilization (OR, 1.01; 95% CI, 0.98-1.05) and readmission (OR, 1.04; 95% CI, 0.99-1.10), but the observed associations did not reach statistical significance.

**Table 2.** Outputs of the Multivariable Model Estimating the Overall Associations of Exit From the Medicare Shared Savings Program With Clinical Quality^a^

Category	Odds ratio (95% CI)					
	HbA_1c_ testing	LDL testing	Eye examination	All diabetes	ED visit	Readmission
ACO exit	0.74 (0.68-0.81)	0.86 (0.76-0.97)	0.93 (0.87-1.00)	0.90 (0.83-0.97)	1.01 (0.98-1.05)	1.04 (0.99-1.10)
Year						
2013	1 [Reference]	1 [Reference]	1 [Reference]	1 [Reference]	1 [Reference]	1 [Reference]
2014	1.04 (0.99-1.09)	0.94 (0.89-0.99)	0.97 (0.93-1.01)	0.97 (0.93-1.01)	1.01 (1.00-1.02)	0.98 (0.95-1.01)
2015	1.16 (1.08-1.25)	0.93 (0.86-1.01)	1.05 (0.97-1.13)	1.04 (0.97-1.12)	1.04 (1.02-1.07)	1.01 (0.97-1.05)
2016	1.22 (1.16-1.29)	0.82 (0.77-0.88)	0.98 (0.94-1.02)	0.95 (0.91-0.99)	1.03 (1.00-1.05)	1.00 (0.97-1.04)
Age group, y						
66-69	1 [Reference]	1 [Reference]	1 [Reference]	1 [Reference]	1 [Reference]	1 [Reference]
70-74	0.99 (0.97-1.02)	1.07 (1.05-1.09)	1.27 (1.25-1.28)	1.20 (1.19-1.22)	1.06 (1.05-1.07)	1.03 (1.01-1.06)
75-79	0.95 (0.91-0.98)	1.04 (1.01-1.08)	1.39 (1.36-1.43)	1.28 (1.25-1.31)	1.25 (1.24-1.26)	1.14 (1.11-1.17)
≥80	NA	NA	NA	NA	1.88 (1.85-1.91)	1.28 (1.25-1.31)
Sex						
Male	1 [Reference]	1 [Reference]	1 [Reference]	1 [Reference]	1 [Reference]	1 [Reference]
Female	1.18 (1.16-1.21)	1.12 (1.10-1.15)	1.28 (1.26-1.30)	1.23 (1.21-1.25)	1.11 (1.10-1.12)	0.96 (0.94-0.97)
Race and ethnicity						
White	1 [Reference]	1 [Reference]	1 [Reference]	1 [Reference]	1 [Reference]	1 [Reference]
Black	0.77 (0.72-0.82)	0.74 (0.70-0.79)	0.85 (0.80-0.89)	0.81 (0.77-0.86)	1.37 (1.34-1.40)	1.21 (1.16-1.25)
Other^b^	1.01 (0.93-1.10)	1.13 (1.02-1.24)	1.01 (0.97-1.05)	1.05 (1.00-1.10)	0.84 (0.77-0.91)	1.05 (1.01-1.10)
Leadership structure						
Hospital-physician partnership	1 [Reference]	1 [Reference]	1 [Reference]	1 [Reference]	1 [Reference]	1 [Reference]
Hospital led	1.11 (1.01-1.23)	1.05 (0.93-1.18)	1.08 (0.95-1.23)	1.09 (0.96-1.23)	0.97 (0.93-1.01)	0.96 (0.92-1.00)
Physician led	1.11 (1.02-1.20)	1.15 (1.04-1.28)	0.99 (0.93-1.05)	1.03 (0.96-1.10)	0.93 (0.90-0.97)	0.98 (0.94-1.02)

Abbreviations: ACO, accountable care organization; ED, emergency department; HbA_1c_, glycated hemoglobin A_1c_; LDL, low-density lipoprotein; NA, not applicable.

^a^ Owing to space constraints, the estimates for some of the ACO characteristics, as well as the hierarchical condition category indicators, have been omitted, but these results are available on request to the corresponding author.

^b^ The Other category includes Asian, Hispanic, Native American, and other or unknown race, as defined by the Centers for Medicare & Medicaid Services’ Beneficiary Race Codes.

**Table 3.** Outputs of the Multivariable Model Estimating Time-Varying Associations of Exit From the Medicare Shared Savings Program With Clinical Quality^a^

Category	Odds ratio (95% CI)					
	HbA_1c_ testing	LDL testing	Eye examination	All diabetes	ED visit	Readmission
Years after ACO exit						
1	0.74 (0.67-0.82)	0.87 (0.77-0.99)	0.94 (0.88-1.01)	0.91 (0.83-0.98)	1.01 (0.97-1.05)	1.05 (0.99-1.11)
2	0.73 (0.62-0.86)	0.80 (0.67-0.96)	0.89 (0.79-1.01)	0.87 (0.76-0.99)	1.03 (0.99-1.08)	1.02 (0.92-1.12)
3	1.31 (0.52-3.31)	0.81 (0.72-0.92)	1.03 (0.85-1.24)	0.95 (0.81-1.11)	1.37 (1.25-1.51)	1.20 (0.97-1.48)
Year						
2013^b^	1 [Reference]	1 [Reference]	1 [Reference]	1 [Reference]	1 [Reference]	1 [Reference]
2014	1.04 (0.99-1.09)	0.94 (0.89-0.99)	0.97 (0.93-1.01)	0.97 (0.93-1.01)	1.01 (0.99-1.02)	0.98 (0.95-1.01)
2015	1.16 (1.08-1.25)	0.93 (0.86-1.01)	1.05 (0.97-1.13)	1.04 (0.97-1.12)	1.04 (1.02-1.07)	1.01 (0.97-1.05)
2016	1.22 (1.16-1.29)	0.82 (0.77-0.88)	0.98 (0.94-1.02)	0.95 (0.91-0.99)	1.03 (1.00-1.05)	1.00 (0.97-1.04)
Age group, y						
66-69	1 [Reference]	1 [Reference]	1 [Reference]	1 [Reference]	1 [Reference]	1 [Reference]
70-74	0.99 (0.97-1.02)	1.07 (1.05-1.09)	1.27 (1.25-1.29)	1.21 (1.19-1.22)	1.06 (1.05-1.07)	1.03 (1.01-1.06)
75-79	0.94 (0.91-0.98)	1.04 (1.01-1.08)	1.40 (1.36-1.43)	1.28 (1.25-1.31)	1.25 (1.24-1.26)	1.14 (1.11-1.17)
≥80	NA	NA	NA	NA	1.88 (1.85-1.91)	1.28 (1.25-1.31)
Sex						
Male	1 [Reference]	1 [Reference]	1 [Reference]	1 [Reference]	1 [Reference]	1 [Reference]
Female	1.18 (1.16-1.21)	1.12 (1.10-1.15)	1.28 (1.26-1.30)	1.23 (1.21-1.25)	1.11 (1.10-1.12)	0.96 (0.94-0.97)
Race and ethnicity						
White	1 [Reference]	1 [Reference]	1 [Reference]	1 [Reference]	1 [Reference]	1 [Reference]
Black	0.77 (0.72-0.82)	0.74 (0.70-0.79)	0.85 (0.80-0.89)	0.81 (0.77-0.86)	1.37 (1.34-1.40)	1.21 (1.16-1.25)
Other^c^	1.01 (0.93-1.10)	1.12 (1.02-1.24)	1.01 (0.97-1.05)	1.05 (1.00-1.10)	0.84 (0.77-0.91)	1.05 (1.01-1.10)
Leadership structure						
Hospital-physician partnership	1 [Reference]	1 [Reference]	1 [Reference]	1 [Reference]	1 [Reference]	1 [Reference]
Hospital led	1.11 (1.01-1.23)	1.05 (0.93-1.18)	1.08 (0.95-1.23)	1.09 (0.96-1.23)	0.97 (0.93-1.00)	0.96 (0.92-1.00)
Physician led	1.11 (1.03-1.20)	1.15 (1.04-1.28)	0.99 (0.93-1.05)	1.03 (0.96-1.10)	0.93 (0.90-0.97)	0.98 (0.94-1.02)

Abbreviations: ACO, accountable care organization; ED, emergency department; HbA_1c_, glycated hemoglobin A_1c_; LDL, low-density lipoprotein; NA, not applicable.

^a^ Owing to space constraints, the estimates for some of the ACO characteristics, as well as the hierarchical condition category indicators, have been omitted, but these results are available on request to the corresponding author.

^b^ This group includes beneficiary-years in Shared Savings Program ACO.

^c^ The Other category includes Asian, Hispanic, Native American, and other or unknown race, as defined by the Centers for Medicare & Medicaid Services’ Beneficiary Race Codes.

**Figure 1.**
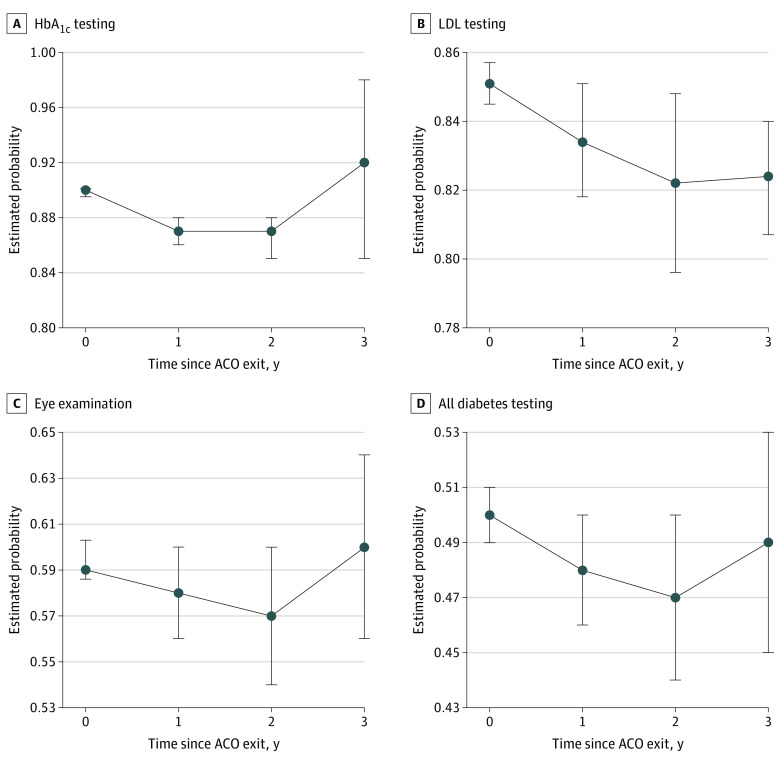
Estimated Probabilities for Annual Preventive Care Services in Years After Accountable Care Organization (ACO) Exit of the Medicare Shared Savings Program The beneficiaries receiving annual preventive care services are 18- to 75-year-old patients with diabetes. The value of 0 indicates the comparison group in the Shared Savings Program, and error bars represent 95% CIs. HbA_1c_ indicates glycated hemoglobin A_1c_; LDL, low-density lipoprotein.

**Figure 2.**
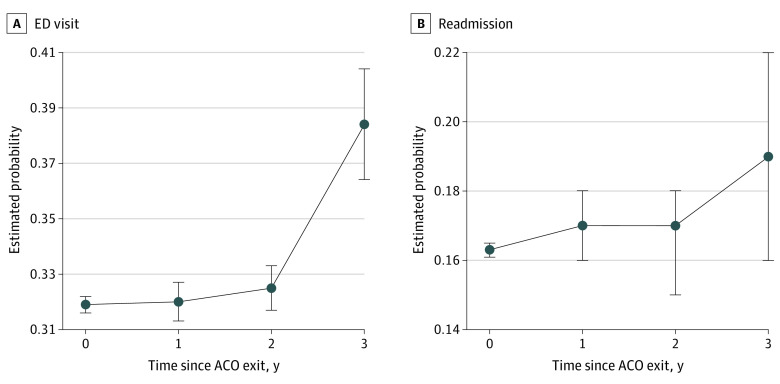
Estimated Probabilities for Hospital Utilization in Years After Accountable Care Organization (ACO) Exit of the Medicare Shared Savings Program The value of 0 indicates the comparison group in the Shared Savings Program, and error bars represent 95% CIs. ED indicates emergency department.

Figures 1 and 2 illustrate the second multivariable model results as marginal probability estimates of the 4 groups, highlighting the time-varying associations that ACO exit had with the rates of annual preventive care services and hospital utilization. Namely, Figure 1 indicates an initial decline in years 1 and 2 for the receipt of annual diabetes complication screening among beneficiaries with diabetes who were aligned to an ACO that exited the SSP but then slightly recovered in year 3. For example, the baseline rate of annual HbA_1c_ testing was 89.8% (95% CI, 89.5%-90.1%) but fell to 86.9% (95% CI, 85.9%-88.0%) and 86.8% (95% CI, 85.0%-88.5%) in years 1 and 2 after exit, respectively, but it then rose to 91.9% (95% CI, 85.3%-98.5%) in year 3. For those measures of hospital utilization, a different pattern emerged, as seen in Figure 2. Specifically, utilization remained stable initially and then rose over time, but the changes were not substantial. This trend was apparent for the frequency of ED visits, the baseline for which was 31.9% (95% CI, 31.6%-32.2%). In years 1 and 2 after exit, it remained relatively stable at 32.0% (95% CI, 31.3%-32.7%) and 32.5% (95% CI, 31.7%-33.3%), respectively, but it rose to 38.4% (95% CI, 36.4%-40.4%) by year 3. The estimation in year 3 had large variability owing to small sample sizes under the unbalanced data structure.

eFigures 1 and 2 in the Supplement present the results of the sensitivity analyses restricting the SSP contract start year to be 2012 and 2013 and separating pre-exit measures from the group of ACOs always staying in the SSP. eFigures 3 and 4 in the Supplement show restriction of the SSP contract start year to between 2012 and 2013 and program exit to 2014. eFigures 5 and 6 in the Supplement show restriction of the SSP contract start year to between 2012 and 2013 and program exit to 2015. The estimated margins of the incidence for the clinical quality measures are included. Generally, dropping out of SSP contracts was associated with reduced preventive services use, except for the group of ACOs exiting the SSP in 2013. The performances of the pre-exit ACOs were similar to those always in the SSP. When checking the confidence intervals, most estimates had large variability owing to small sample sizes. Therefore, it was reasonable to combine the pre-exit measures with those of ACOs always in the SSP.

When models were refitted and the sample restricted by other years of the SSP contract initiation, the primary findings on the SSP exit associations were unchanged, suggesting no statistically significant cohort effects. When the additional control group of ACO-unaligned beneficiaries was included, the conclusions on the exit association evaluation remained the same, though the estimated probabilities might differ for incidences of preventive care services. The resulted incidence estimates are presented in eFigures 7 and 8 in the Supplement.

Finally, when models were refitted and interactions introduced between the exposure variables and dummy indicators of year to allow different comparison groups to have different nonlinear increments over time, the coefficient estimates of the interaction terms were not statistically significantly different from each other, supporting that the primary findings were robust.

## Discussion

This study had 2 principal findings. First, beneficiaries aligned to an ACO that exited the SSP received poorer quality of care after exit than their counterparts aligned to an ACO that stayed in the program. Second, the associations of ACO exit with the quality of care received were time varying. In particular, we observed a V-shaped recovery for receipt of preventive services among beneficiaries with diabetes (ie, annual HbA_1c_ testing, LDL cholesterol testing, all diabetes complication screening), whereas hospital utilization (ie, rates of ED visit, readmission) tended to be stable.

The peer-reviewed literature on ACOs exiting the SSP is limited to 1 recent study from Bleser and colleagues,^2^ who noted that program exits decreased after ACOs’ third year. Factors associated with longer survival included shared-savings bonus payment achievement, more care coordination, higher financial performance benchmarks, market-level Medicare cost growth, lower-risk patients, and contracts without downside risk. To our knowledge, the present study represents the first to characterize the associations of ACO exit with beneficiary health. These findings indicate that the benefits of SSP participation, particularly around preventive health measures,^10,11^ quickly dissipate following program exit. One potential explanation is that the care transformation processes, which are implemented following an organization’s decision to join the SSP, are not “hardwired.” Without the potential for shared savings, health care professional groups have little incentive to maintain the data systems, analytic support, and coordination capabilities that are needed to support these processes.

### Limitations

This study has several limitations that merit discussion. First, we must acknowledge that the decision to exit the SSP is nonrandom and may be endogenous with organizational factors that could affect care delivery and outcomes. Second, the health care professional groups in this sample may have participated in other federal, state, and commercial programs (eg, Comprehensive Primary Care initiative) concurrent with the SSP, which could have affected their clinical performance. However, for such participation to bias the present results, it would have to differ systematically between groups in exiting and nonexiting ACOs. Third, our approach could be compromised by “shocks” independent of program exit that may have decreased preventive services use and increased hospital utilization. To combat this validity threat, we conducted a comprehensive set of sensitivity analyses that included an additional control group of ACO-unaligned beneficiaries. This allowed us to difference out changes in outcomes that would have occurred in the absence of the SSP.

Despite these limitations, this study has important policy implications. Namely, it suggests that the care redesign, which occurs during program participation, is fragile. This may be because of the fact that the SSP’s incentives around quality are too weak, which is relevant given CMS’s recent major overhaul to the program, known as Pathways to Success. Under Pathways to Success, current ACOs are required to shift to downside risk in as little as 1 year.^12^ However, requiring participating ACOs to shift too quickly to downside risk may affect SSP participation. Specifically, the Pathways to Success announcement came on the heels of a survey conducted by the National Association of ACOs that targeted ACO administrators. More than 70% of respondents indicated that they would leave the SSP if forced to take on downside risk.^13^ Indeed, 40% of SSP ACOs that faced contract renewal in July 2019 chose to exit the program instead.^14^ The present analysis demonstrates the potentially damaging effects on beneficiary health that a rapid transition to downside risk could have.

## Conclusions

In this cohort study, we found that beneficiaries who were aligned to an exiting ACO received poorer quality of care after exit than their counterparts aligned to an ACO, which remained in the SSP. Further research, synthesizing both quantitative (such as the present study) and qualitative analyses of organizational decision-making around program participation, is needed to help inform health policy interventions that encourage organizations to stay on their path to accountable care.

## Notes

Supplement.
eMethods.
**eFigure 1.** Sensitivity Analyses for the Pre- and Post-Exit Comparison and Cohort Effect on Rates of Receiving Annual Preventive Care Services. **eFigure 2.** Sensitivity Analyses for the Pre- and Post-Exit Comparison and Cohort Effect on Rates of Hospital Utilization. **eFigure 3.** Sensitivity Analyses for the Pre- and Post-Exit Comparison and Cohort Effect. **eFigure 4.** Sensitivity Analyses for the Pre- and Post-Exit Comparison and Cohort Effect. **eFigure 5.** Sensitivity Analyses for the Pre- and Post-Exit Comparison and Cohort Effect. **eFigure 6.** Sensitivity Analyses for the Pre- and Post-Exit Comparison and Cohort Effect. **eFigure 7.** Sensitivity Analyses for the Time-Varying Effect Estimation Model for Rates of Receiving Annual Preventive Care Services: Comparing Results from the Main Analysis to an Analysis that Includes a Control Group of Accountable Care Organization (ACO)-Unaligned Beneficiaries and to an Analysis that Separates ACOs into Two Separate Cohorts Based on Contract Start Date (2012 to 2013 versus 2013 to 2014). **eFigure 8.** Sensitivity Analyses for the Time-Varying Effect Estimation Model for Rates of Hospital Utilization: Comparing Results from the Main Analysis to an Analysis that Includes a Control Group of Accountable Care Organization (ACO)-Unaligned Beneficiaries and to an Analysis that Separates ACOs into Two Separate Cohorts Based on Contract Start Date (2012 to 2013 versus 2013 to 2014).
