# Error in Funding/Support

**DOI:** 10.1001/jamahealthforum.2022.1390

**Published:** 2022-05-20

In the Original Investigation titled, “Use of Preventive Care Services and Hospitalization Among Medicare Beneficiaries in Accountable Care Organizations That Exited the Shared Savings Program,”^1^ published online January 7, 2022, there were missing entries for coauthor John M. Hollingsworth, MD, MS, that should have been included in the Funding/Support section. Dr Hollingsworth is supported by the Agency for Healthcare Research and Quality through grants R01HS024728 and R01HS024525. The article has been corrected.
