# Supplementary material for: Use of Preventive Care Services and Hospitalization Among Medicare Beneficiaries in Accountable Care Organizations That Exited the Shared Savings Program
Source: JAMA Health Forum. 2022 Jan 7;3(1):e214452. doi: 10.1001/jamahealthforum.2021.4452 (PMC8765717; doi:10.1001/jamahealthforum.2021.4452)
Supplement: Supplement. — eMethods. eFigure 1. Sensitivity Analyses for the Pre- and Post-Exit Comparison and Cohort Effect on Rates of Receiving Annual Preventive Care Services. eFigure 2. Sensitivity Analyses for the Pre- and Post-Exit Comparison and Cohort Effect on Rates of Hospital Utilization. eFigure 3. Sensitivity Analyses for the Pre- and Post-Exit Comparison and Cohort Effect. eFigure 4. Sensitivity Analyses for the Pre- and Post-Exit Comparison and Cohort Effect. eFigure 5. Sensitivity Analyses for the Pre- and Post-Exit Comparison and Cohort Effect. eFigure 6. Sensitivity Analyses for the Pre- and Post-Exit Comparison and Cohort Effect. eFigure 7. Sensitivity Analyses for the Time-Varying Effect Estimation Model for Rates of Receiving Annual Preventive Care Services: Comparing Results from the Main Analysis to an Analysis that Includes a Control Group of Accountable Care Organization (ACO)-Unaligned Beneficiaries and to an Analysis that Separates ACOs into Two Separate Cohorts Based on Contract Start Date (2012 to 2013 versus 2013 to 2014). eFigure 8. Sensitivity Analyses for the Time-Varying Effect Estimation Model for Rates of Hospital Utilization: Comparing Results from the Main Analysis to an Analysis that Includes a Control Group of Accountable Care Organization (ACO)-Unaligned Beneficiaries and to an Analysis that Separates ACOs into Two Separate Cohorts Based on Contract Start Date (2012 to 2013 versus 2013 to 2014). [file jamahealthforum-e214452-s001.pdf]

Supplemental Online Content

Si Y, Moloci N, Murali S, Krein S, Ryan A, Hollingsworth JM. Use of preventive care services and hospitalization among Medicare beneficiaries in accountable care organizations that exited the Shared Savings Program. *JAMA Health Forum*. 2022;3(1):e214452. doi:10.1001/jamahealthforum.2021.4452

eMethods.
eFigure 1. Sensitivity Analyses for the Pre- and Post-Exit Comparison and Cohort Effect on Rates of Receiving Annual Preventive Care Services.
eFigure 2. Sensitivity Analyses for the Pre- and Post-Exit Comparison and Cohort Effect on Rates of Hospital Utilization.
eFigure 3. Sensitivity Analyses for the Pre- and Post-Exit Comparison and Cohort Effect.
eFigure 4. Sensitivity Analyses for the Pre- and Post-Exit Comparison and Cohort Effect.
eFigure 5. Sensitivity Analyses for the Pre- and Post-Exit Comparison and Cohort Effect.
eFigure 6. Sensitivity Analyses for the Pre- and Post-Exit Comparison and Cohort Effect.
eFigure 7. Sensitivity Analyses for the Time-Varying Effect Estimation Model for Rates of Receiving Annual Preventive Care Services: Comparing Results from the Main Analysis to an Analysis that Includes a Control Group of Accountable Care Organization (ACO)-Unaligned Beneficiaries and to an Analysis that Separates ACOs into Two Separate Cohorts Based on Contract Start Date (2012 to 2013 versus 2013 to 2014).
eFigure 8. Sensitivity Analyses for the Time-Varying Effect Estimation Model for Rates of Hospital Utilization: Comparing Results from the Main Analysis to an Analysis that Includes a Control Group of Accountable Care Organization (ACO)-Unaligned Beneficiaries and to an Analysis that Separates ACOs into Two Separate Cohorts Based on Contract Start Date (2012 to 2013 versus 2013 to 2014).

This supplemental material has been provided by the authors to give readers additional information about their work.

© 2022 Si Y et al. *JAMA Health Forum.*

eMethods

Sensitivity Analyses

First, recognizing the possibility of cohort effects and trend heterogeneity, we refined our models, restricting the sample by the SSP contract initiation and end years and separating the pre-exit measures from the observations in the group that always stayed in the SSP. Second, we re-estimated the association between SSP exit and each of our outcomes, including the other control group of ACO-unaligned beneficiaries to approximate outcomes that would have occurred in the absence of the SSP. This included all alignment-eligible beneficiaries who were unaligned to any ACO during the period after the SSP's launch in 2012. We used CMS's SSP alignment algorithm to attribute these beneficiaries to a non-ACO Tax Identification Number.10 Finally, from an exploratory analysis, we found evidence to suggest non-linear trends for some of our outcomes across years and different ACO groups. Therefore, we repeated our analysis, introducing interactions between exposure variables and dummy indicators of the time of year such that different comparison groups would have different, non-linear increments over time.

© 2022 Si Y et al. *JAMA Health Forum.*

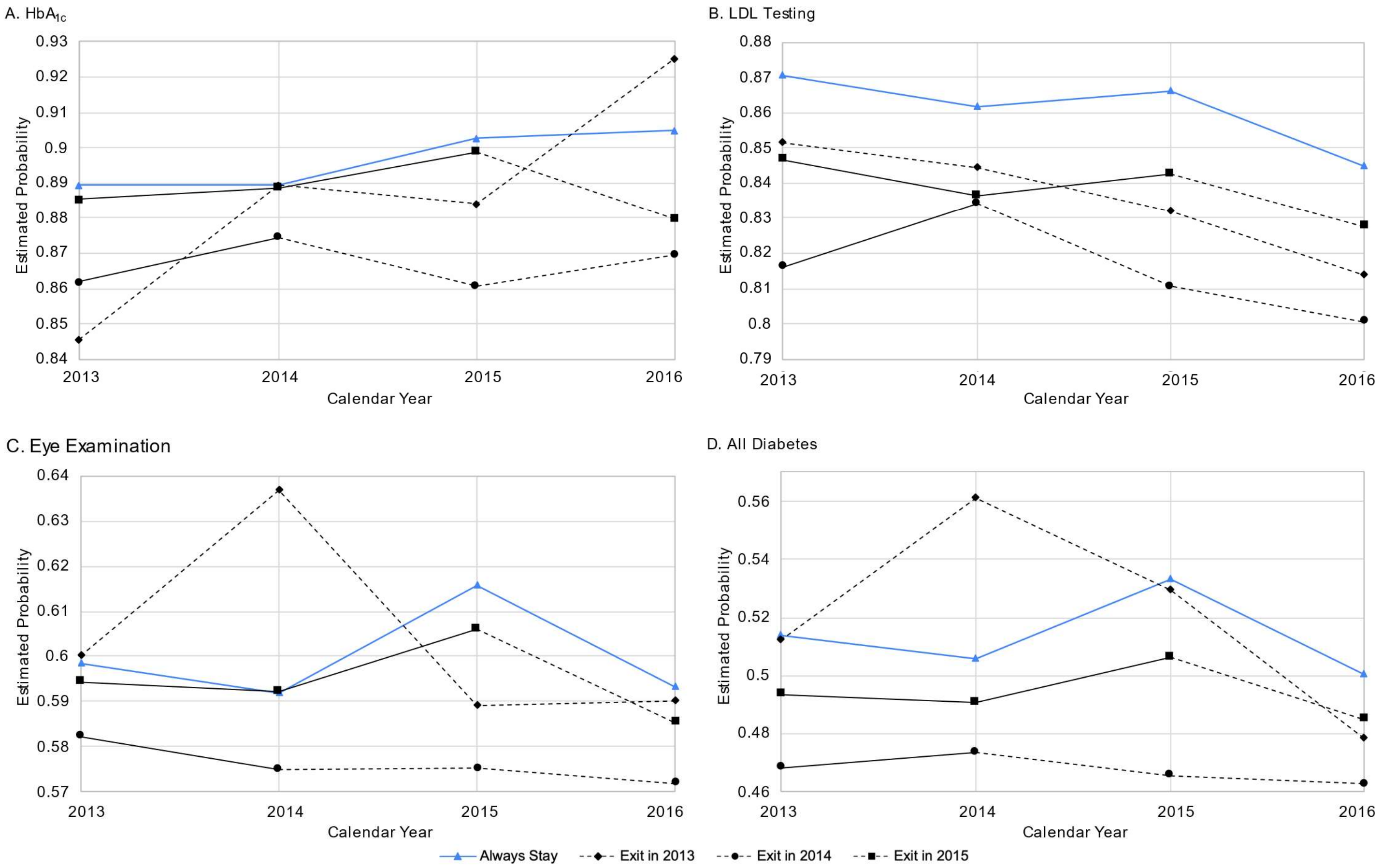

eFigure 1. Sensitivity Analyses for the Pre- and Post-Exit Comparison and Cohort Effect on Rates of Receiving Annual Preventive Care Services.
The cohort is restricted to Accountable Care Organizations with contract start dates between 2012 and 2013.

Abbreviations: HbA_{1c}, glycated hemoglobin testing; LDL, low-density lipoprotein.

Note: Estimated marginal probabilities are displayed for four groups of Accountable Care Organizations, including those always staying in the Shared Savings Program and those three groups of dropouts differentiated by the year of the Shared Savings Program exit. The solid lines indicated the pre-exit measures.

© 2022 Si Y et al. *JAMA Health Forum.*

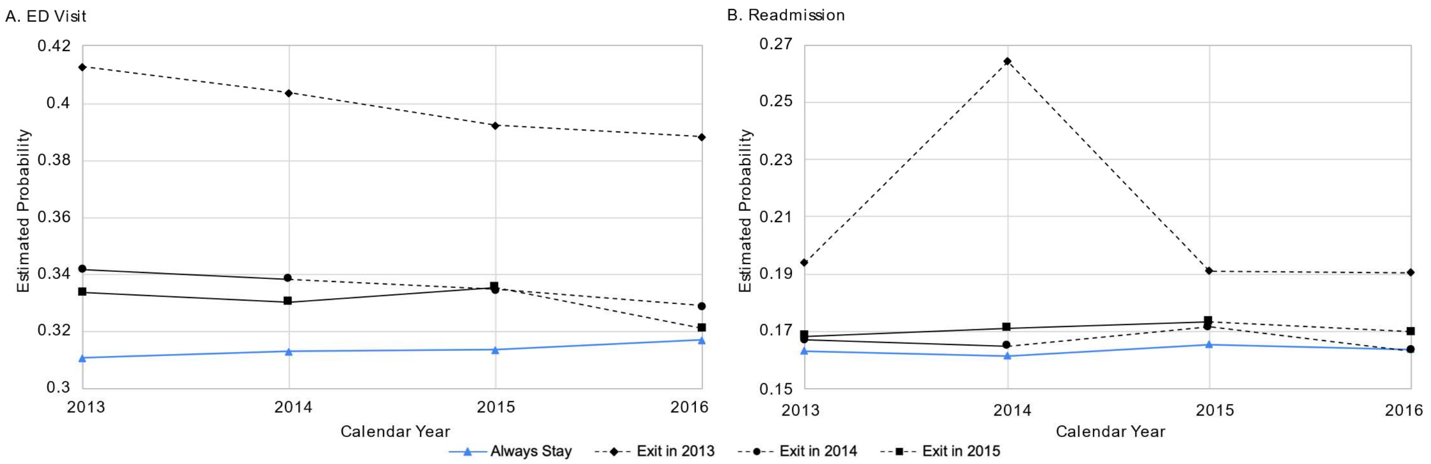

eFigure 2. Sensitivity Analyses for the Pre- and Post-Exit Comparison and Cohort Effect on Rates of Hospital Utilization.
The cohort is restricted to Accountable Care Organizations with contract start dates between 2012 and 2013.

Abbreviations: ED, emergency department

Note: Estimated marginal probabilities are displayed for four groups of Accountable Care Organizations, including those always staying in the Shared Savings Program and those three groups of dropouts differentiated by the year of the Shared Savings Program exit. The solid lines indicated the pre-exit measures.

© 2022 Si Y et al. *JAMA Health Forum.*

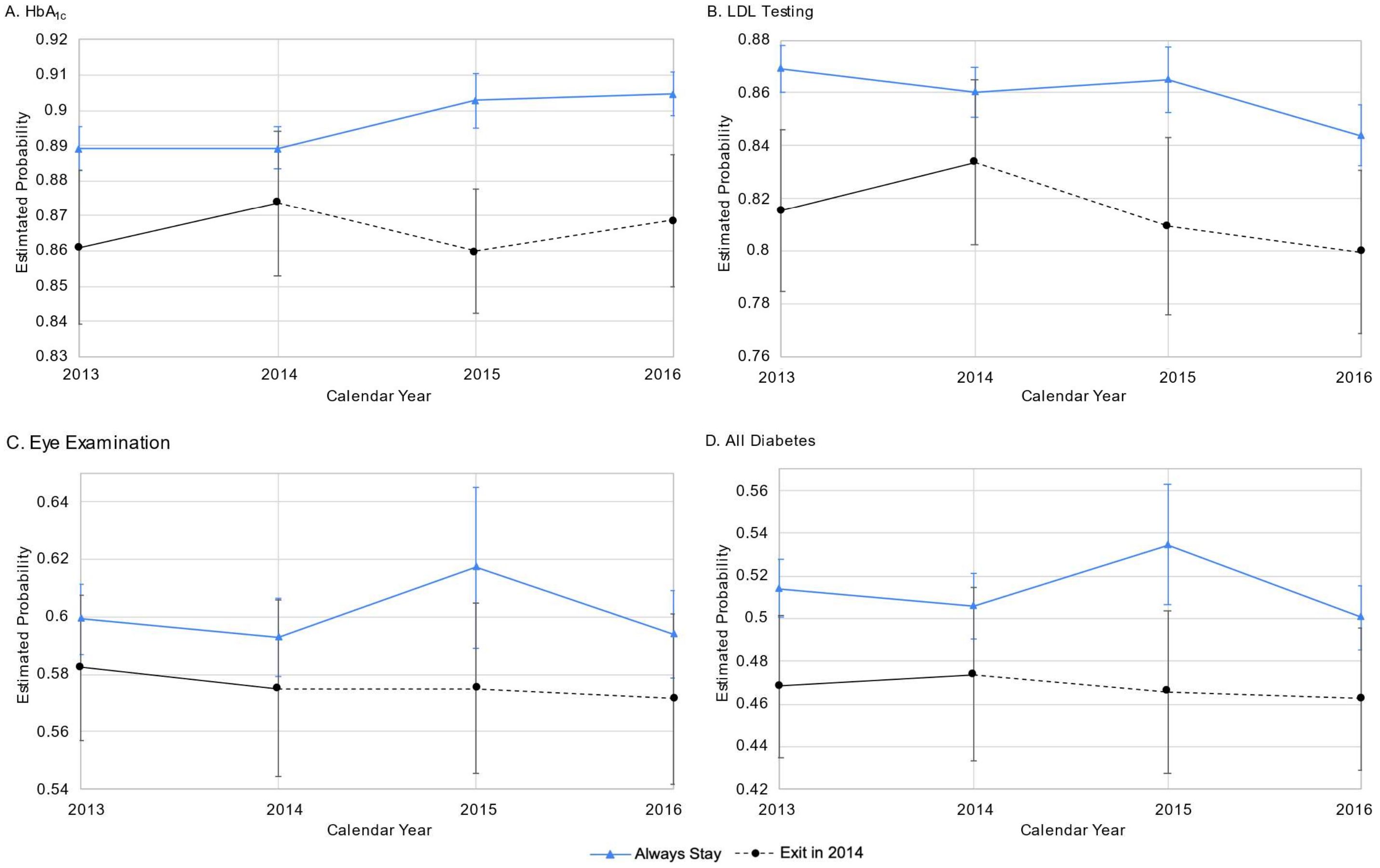

eFigure 3. Sensitivity Analyses for the Pre- and Post-Exit Comparison and Cohort Effect. The cohort is restricted to Accountable Care Organizations with contract start dates between 2012 and 2013 and program exit in 2014.

Abbreviations: HbA_{1c}, glycated hemoglobin testing; LDL, low-density lipoprotein.

Note: Estimated marginal probabilities with corresponding 95% confidence intervals are displayed for two groups of Accountable Care Organizations, including those always staying in the Shared Savings Program and the group exit the Shared Savings Program in 2014. The solid lines indicated the pre-exit measures.

© 2022 Si Y et al. *JAMA Health Forum.*

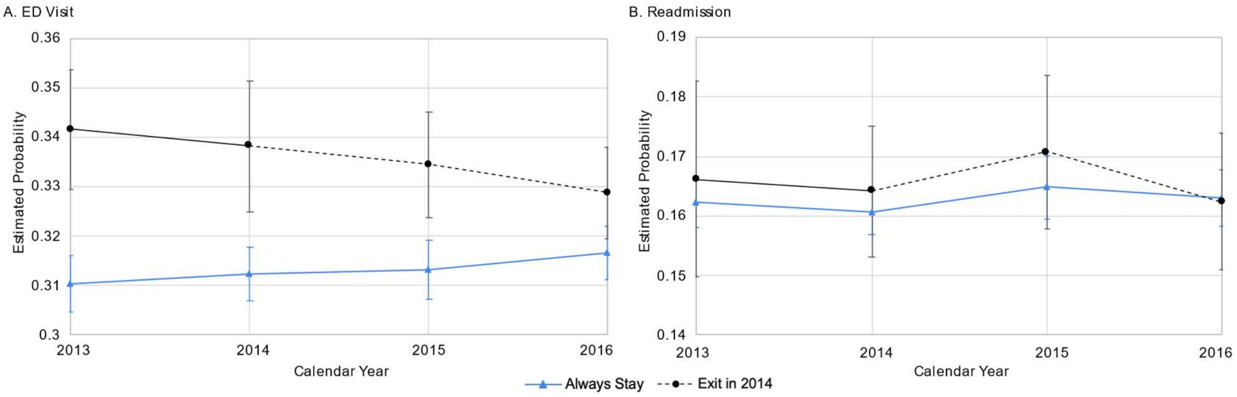

eFigure 4. Sensitivity Analyses for the Pre- and Post-Exit Comparison and Cohort Effect. The cohort is restricted to Accountable Care Organizations with contract start dates between 2012 and 2013 and program exit in 2014.

Abbreviations: ED, emergency department

Note: Estimated marginal probabilities with corresponding 95% confidence intervals are displayed for two groups of Accountable Care Organizations, including those always staying in the Shared Savings Program and the group exit the Shared Savings Program in 2014. The solid lines indicated the pre-exit measures.

© 2022 Si Y et al. *JAMA Health Forum.*

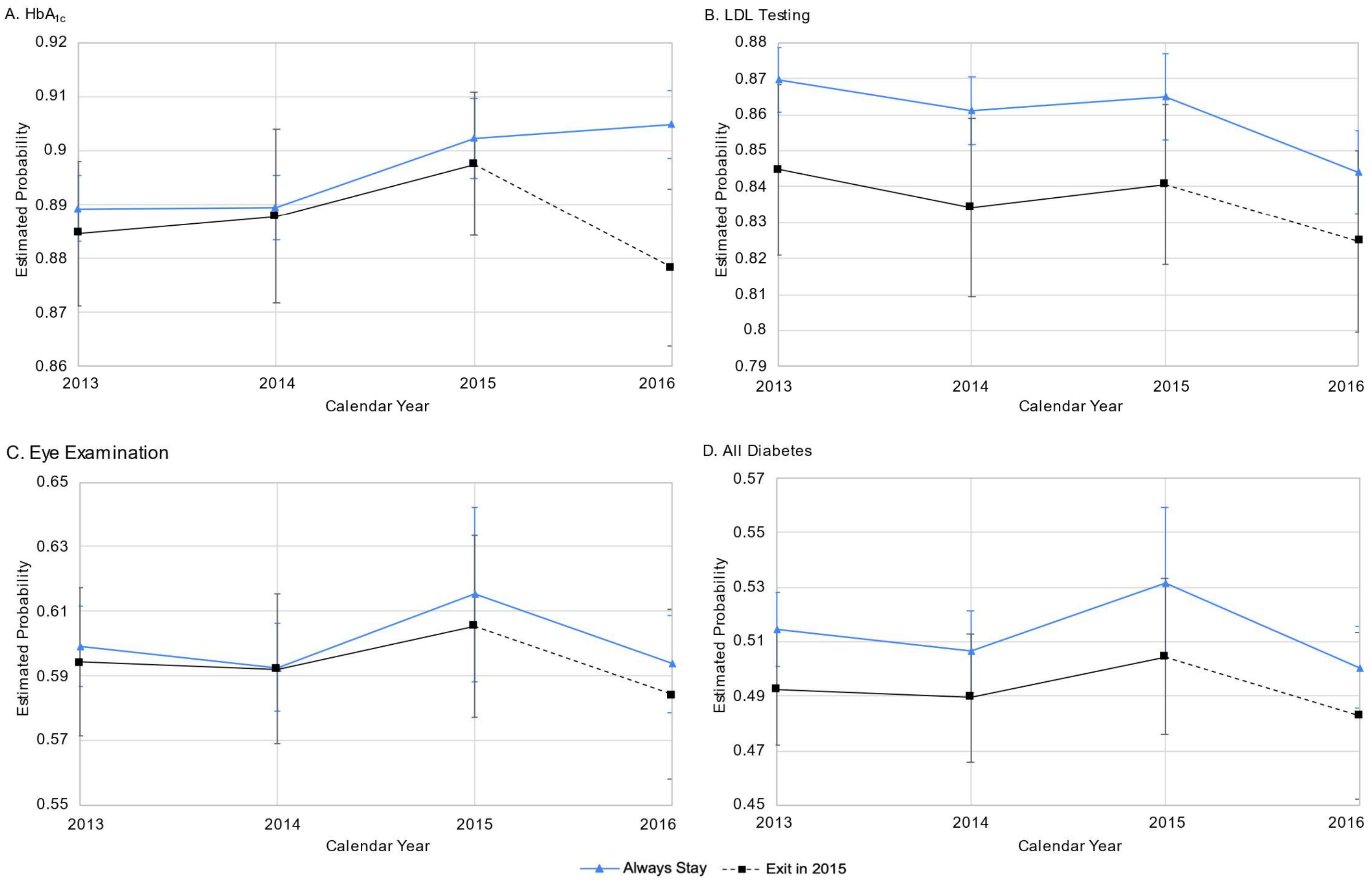

eFigure 5. Sensitivity Analyses for the Pre- and Post-Exit Comparison and Cohort Effect. The cohort is restricted to Accountable Care Organizations with contract start dates between 2012 and 2013 and program exit in 2015.

Abbreviations: HbA_{1c}, glycated hemoglobin testing; LDL, low-density lipoprotein.

Note: Estimated marginal probabilities with corresponding 95% confidence intervals are displayed for two groups of Accountable Care Organizations, including those always staying in the Shared Savings Program and the group exit the Shared Savings Program in 2015. The solid lines indicated the pre-exit measures.

© 2022 Si Y et al. *JAMA Health Forum.*

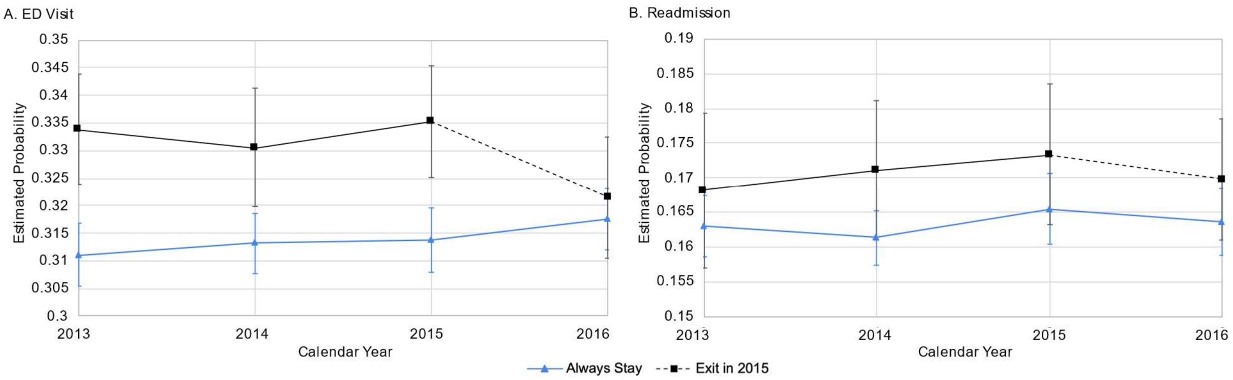

eFigure 6. Sensitivity Analyses for the Pre- and Post-Exit Comparison and Cohort Effect. The cohort is restricted to Accountable Care Organizations with contract start dates between 2012 and 2013 and program exit in 2015.

Abbreviations: ED, emergency department

Note: Estimated marginal probabilities with corresponding 95% confidence intervals are displayed for two groups of Accountable Care Organizations, including those always staying in the Shared Savings Program and the group exit the Shared Savings Program in 2015. The solid lines indicated the pre-exit measures.

© 2022 Si Y et al. *JAMA Health Forum.*

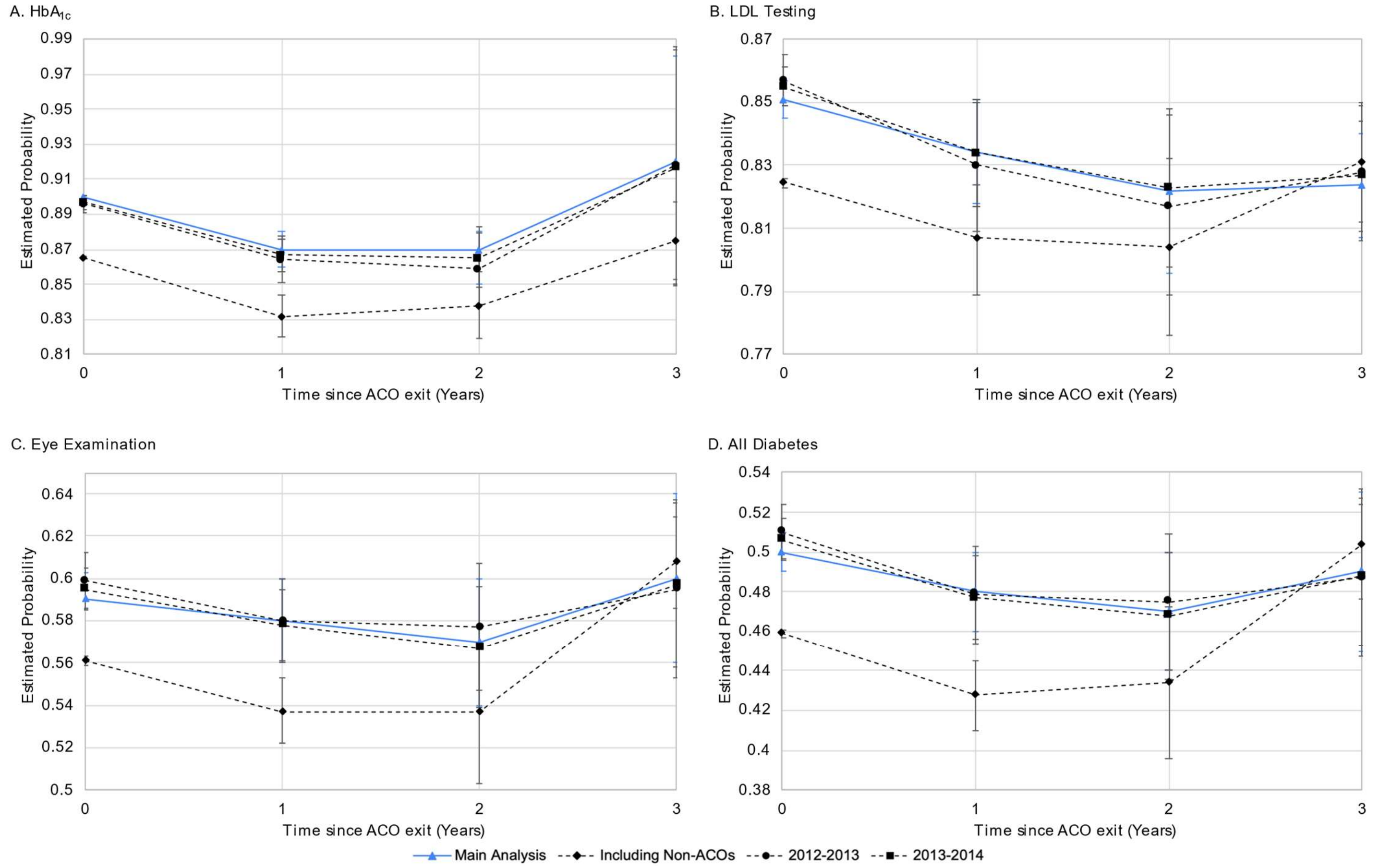

eFigure 7. Sensitivity Analyses for the Time-Varying Effect Estimation Model for Rates of Receiving Annual Preventive Care Services: Comparing Results from the Main Analysis to an Analysis that Includes a Control Group of Accountable Care Organization (ACO)-Unaligned Beneficiaries and to an Analysis that Separates ACOs into Two Separate Cohorts Based on Contract Start Date (2012 to 2013 versus 2013 to 2014).

Abbreviations: HbA_{1c}, glycated hemoglobin testing; LDL, low-density lipoprotein.

Note: Estimated marginal probabilities with corresponding 95% confidence intervals are displayed in years after the Shared Savings Program exit.

© 2022 Si Y et al. *JAMA Health Forum.*

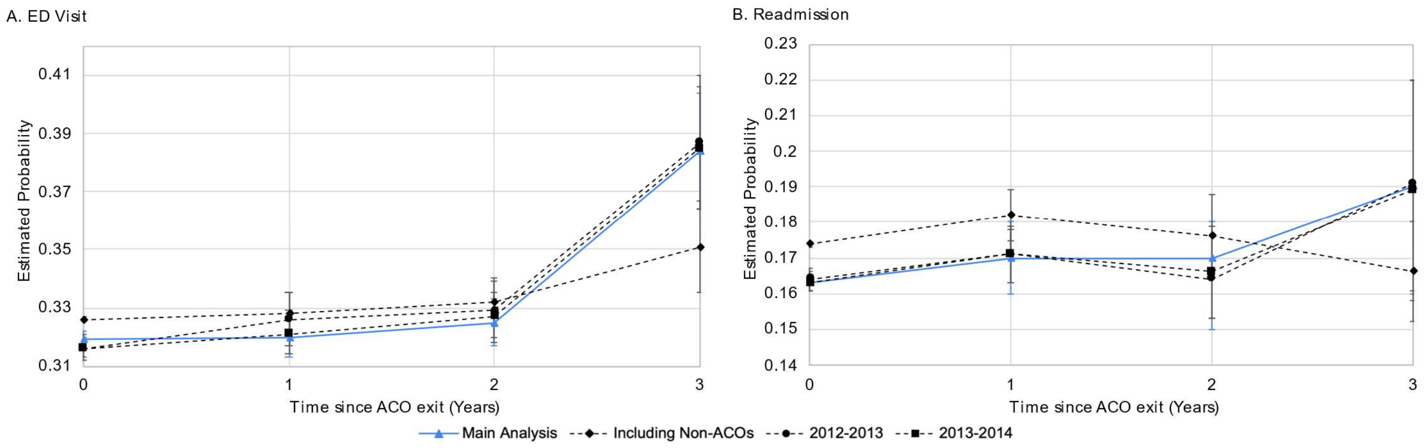

eFigure 8. Sensitivity Analyses for the Time-Varying Effect Estimation Model for Rates of Hospital Utilization: Comparing Results from the Main Analysis to an Analysis that Includes a Control Group of Accountable Care Organization (ACO)-Unaligned Beneficiaries and to an Analysis that Separates ACOs into Two Separate Cohorts Based on Contract Start Date (2012 to 2013 versus 2013 to 2014).

Abbreviations: ED, emergency department.

Note: Estimated marginal probabilities with corresponding 95% confidence intervals are displayed in years after the Shared Savings Program exit.

© 2022 Si Y et al. *JAMA Health Forum.*
